# Validation of Medicare Rehabilitation Functional Assessments in Routine Care

**DOI:** 10.1001/jamanetworkopen.2020.4702

**Published:** 2020-05-13

**Authors:** Kevin A. Kerber, Lesli E. Skolarus, Chunyang Feng, James F. Burke

**Affiliations:** 1 Department of Neurology, University of Michigan, Ann Arbor; 2 Department of Neurology, Ann Arbor Veteran’s Affairs, Ann Arbor, Michigan

## Abstract

**Importance:**

Assessment of functional outcomes is currently limited by a lack of large data sets. Functional assessments are included in Medicare rehabilitation assessment files, yet the validity of these measures in routine care is unknown.

**Objective:**

To evaluate the validity of individual-level routine care functional assessments in Medicare rehabilitation settings compared with criterion-standard National Health and Aging Trends Study (NHATS) research assessments obtained no more than 90 days later.

**Design, Setting, and Participants:**

This cross-sectional study of individuals aged 65 years and older used the 2011 to 2015 NHATS linked with Medicare assessment files. Individuals with a discharge assessment from inpatient rehabilitation facilities, skilled nursing facilities, or home health agencies and a criterion-standard NHATS assessment were included. Data analysis was performed June 2019 to November 2019.

**Main Outcomes and Measures:**

Summary functional assessment based on independence with eating, toilet hygiene, bathing, dressing, transfers, and mobility or walking. Linear regression was used to assess agreement between the 2 scales, adjusting for time between assessments and rehabilitation setting.

**Results:**

A total of 1036 adults aged 65 years and older (671 [64.8%] aged ≥80 years; 670 [64.7%] women; 685 [66.1%] white participants) met the study criteria. The correlation of the assessments was 0.63 (95% CI, 0.59 to 0.66; mean [SD] rehabilitation score, 27.5 [7.2]; mean [SD] NHATS score, 30.5 [10.1]). The correlation increased to 0.66 (95% CI, 0.60 to 0.71) for assessments no more than 30 days apart. The linear regression model adjusting for rehabilitation setting and days between evaluations found the assessments were strongly correlated (β = 1.00 [95% CI, 0.93 to 1.08]; intercept, 0.72 [95% CI, −1.79 to 3.24]; *R*^2^ = 0.42). Differences in scores were generally small (mean [SD] of NHATS − rehabilitation score, 2.96 [7.91]), and only 59 assessments (5.7%) differed by more than 2 SDs of the mean difference. Rehabilitation service scores were typically higher than NHATS scores in individuals with lower mean scores; however, the population with lower mean scores was small (156 [15.1%]).

**Conclusions and Relevance:**

In this large sample of older US adults, routine care rehabilitation facility functional assessments had overall moderate correlation with criterion-standard research assessments.

## Key Points

**Question:**

Are functional assessments in rehabilitation settings valid measures?

**Findings:**

In this cross-sectional study of 1036 Medicare beneficiaries, the correlation of functional assessments from rehabilitation services with National Health and Aging Trends Study assessments was 0.63 when assessments were performed no more than 90 days apart and 0.66 when performed no more than 30 days apart. Differences in assessment scores were generally small; however, assessments from the National Health and Aging Trends Study tended to be lower than those from rehabilitation services in a small sample of older adults with less function.

**Meaning:**

These findings suggest that Medicare rehabilitation functional assessments may be sufficiently associated with functional outcomes for use in some research applications.

## Teaser

This cross-sectional study evaluates the validity of individual-level routine care functional assessments in Medicare rehabilitation settings compared with criterion-standard National Health and Aging Trends Study research assessments obtained no more than 90 days later.

## Introduction

Function is an important patient-centered outcome, but it is difficult to assess on a national scale. Medicare beneficiaries undergo functional assessments while receiving rehabilitation services from inpatient rehabilitation facilities (IRFs), skilling nursing facilities (SNFs), and home health agencies (HHAs). The assessments are based on the individual’s ability to perform specific activities, as measured by rehabilitation staff. While the purpose of the assessments is to estimate resource needs and determine payment amounts, these data are also available for use in research studies. However, the validity of the assessments performed in the rehabilitation setting is not known.

The assessment instruments used in rehabilitation settings are the Functional Independence Measure (FIM), Minimum Data Set, and the Outcome and Assessment Information Set (eAppendix and eTable 1 in the Supplement). These instruments overlap in the activities and scales used to rate functioning. Prior studies support the reliability and validity of these instruments.^1,2,3,4,5,6,7^ However, prior studies were generally performed in small samples of patients and with a limited number of evaluators who received specific training. To our knowledge, nationally representative validation of routine care assessments has not been performed. Because of the lack of large-scale validation, concerns have been raised about the appropriateness of using the data for research purposes.^3,7^ These concerns are based on the large number of staff who perform the assessments, the lack of required standardized training on evaluation and scoring, and a potential bias because of facility performance and associated payments.

The validity of Medicare rehabilitation assessments can be explored by comparing them with criterion-standard functional assessments with known reliability and validity,^8,9^ such as those obtained as part of the National Health and Aging Trends Study (NHATS), a large population-based survey of health and functional ability trends among Medicare beneficiaries aged 65 years and older.^10^ The NHATS is also linked to Medicare, creating the opportunity to compare functional assessments from rehabilitation settings with research-based functional assessments. The advantages of this comparison are that the data are drawn from a large, nationally representative sample in routine practice, masked to any knowledge of a subsequent comparison with a criterion standard that is complete and rigorous. Disadvantages of this comparison are the variable time interval between Medicare and NHATS assessments as well as differences in the instruments used by the various rehabilitation settings.

In this study, we aimed to compare the functional assessments performed in routine care Medicare rehabilitation settings with the NHATS assessments considered to be the criterion standard. To enable the comparison, we crosswalked overlapping functional instruments and limited the comparisons to NHATS assessments performed no more than 90 days after an assessment in a rehabilitation setting. If the correlation and agreement between the assessments are satisfactory, they would support the use of Medicare rehabilitation functional assessments in some research contexts.

## Methods

### Study Design and Setting

The design was a cross-sectional validation study using retrospective data comparing functional assessments in Medicare rehabilitation settings with similar criterion-standard assessments from the NHATS. The setting was Medicare rehabilitation facilities (IRFs and SNFs), and older adults’ homes for HHA and NHATS assessments. The study was approved by the University of Michigan institutional review board, and written informed consent was obtained from NHATS participants at the time of enrollment in NHATS. This study followed the Strengthening the Reporting of Observational Studies in Epidemiology (STROBE) reporting guideline.

### Data Sources

We used the 2011 to 2015 NHATS linked with Medicare files. The data were linked by the Centers for Medicare & Medicaid Services. Rehabilitation functional assessment data from IRFs, SNFs, or HHAs were obtained from Medicare functional assessment files. The NHATS is a population-based survey, with oversampling of the oldest population and African American participants. Trained staff perform annual in-person data collection from participants regarding their physical and cognitive function, social environment, and participation in daily activities. Detailed methods of the NHATS have been published previously.^8,11^

### Study Population

The inclusion criteria were NHATS participants with IRF, SNF, or HHA discharge rehabilitation claims no more than 90 days before the NHATS assessment. We excluded assessments when the participant was in a nursing home at the time of NHATS assessment because self-care activities are not obtained in this setting. We also excluded assessments when a hospitalization occurred in the period between the rehabilitation assessment and the NHATS assessment because we anticipated that the hospitalization could lead to a change in function that would not be captured by the assessments. When an individual had more than 1 eligible assessment, we used the assessment closest in time to the subsequent NHATS assessment, regardless of setting. Race/ethnicity was self-reported using options defined by NHATS.

### Variables

There are different but overlapping functional assessment instruments for each rehabilitation setting, ie, the FIM, the Minimum Data Set, and the Outcome and Assessment Information Set (eTable 1 in the Supplement). We identified 6 functional capacity instrument components based on similar domains and similar scoring scales in both NHATS and the rehabilitation assessments. The overlapping components were eating, toilet hygiene, bathing, dressing, bed transfers, and mobility or walking. For each component, the scores indicated whether help (from a device or another person) was required to perform the activity and the extent of any help required, ranging from completely independent to completely dependent.

The questions used in the instruments were directly comparable for eating, toilet hygiene, and bathing. For the other domains, there were some differences in both the specific domains and how they were queried. Despite these differences, we included these domains because we concluded it was likely that the elements were sufficiently similar to capture the key underlying constructs. For bed transfers, the NHATS questions asked about transitions from bed, whereas the FIM question asked about transitions from bed, chairs, or wheelchairs. The dressing activity variable in NHATS does not specify upper or lower body, whereas the FIM uses separate variables for upper and lower body dressing. We used the most dependent FIM dressing score for the comparison, as done in previous research.^12^ For mobility and walking functional capacity, we used the NHATS mobility outside questions and the FIM’s locomotion assessment. Item scoring by rehabilitation setting appears in eTable 2, eTable 3, and eTable 4 in the Supplement. Each FIM item is scored from 1 to 7, with higher scores representing greater function. Based on our 6 FIM components, the overall functional score ranged from 7 to 49.

For rehabilitation setting assessments, staff score each item based on observed abilities during the previous 1 to 7 days. For NHATS, trained interviewers ask participants to self-report the level of function for each activity over the last month.^8,10,13^ For each item, individuals are first asked if they require a device to perform the activity. Next, individuals are asked if anyone has helped them with the activity in the last month. Individuals who report help are then asked how often they performed the activity by themselves and without help. Variables in NHATS were converted to a 7-point scale to match the FIM scale (eTable 2, eTable 3, and eTable 4 in the Supplement).

To calculate the days between the rehabilitation assessment and the NHATS assessment, we used the date of the rehabilitation assessment and the fifteenth day of the month for the NHATS assessment. The fifteenth day of the month was used for the NHATS date because NHATS only reports the month and year of the assessment.

### Primary and Secondary Outcomes

The primary outcome was the overall function score. This was calculated by summing the scores for the 6 individual activities in each rehabilitation setting and NHATS. Secondary outcomes were the individual components of the overall function score.

### Statistical Analysis

We used descriptive statistics to summarize the population, days between assessments, and the function scores. We compared the correlation of the overall function score between Medicare functional assessments and NHATS assessments using the Pearson correlation coefficient for eligible assessments within 90 days and separately for assessments within 30 and 15 days. We used linear regression to examine the association of the rehabilitation facility score with the NHATS score, the intercept, and the squared value of the correlation coefficient (ie, *R*^2^) for the rehabilitation setting and NHATS assessment, adjusting for days between the assessments. Next, we added a variable of rehabilitation facility setting to assess differences explained by rehabilitation setting. We calculated the difference in assessment scores (NHATS score − rehabilitation service score) and used Bland-Altman difference against the mean plots to assess agreement between the 2 assessments and to visually inspect for variation in the differences in scores across the range of mean scores.^14^ Items with missing data were infrequent (ie, <1% per item) and therefore excluded. All analyses were performed using Stata version 15.1 (StataCorp) and SAS statistical software version 9.4 (SAS Institute). Data analyses were performed from June 2019 to November 2019. Statistical significance was set at α < .05, and all tests were 2-tailed.

## Results

### Study Population

From 2011 to 2015, we identified 6436 NHATS assessments that matched Medicare rehabilitation setting functional assessments. After excluding NHATS assessments that occurred 90 days or more after the rehabilitation assessment, assessments with an interim hospitalization, and multiple assessments per individual, our final study population included 1036 individuals (eFigure 1 in the Supplement). Characteristics of the study population are presented in Table 1. In the cohort, 671 participants (64.8%) were aged 80 years or older, 670 (64.7%) were women, and 685 (66.1%) were white patients. Overall, 27 participants (2.6%) used IRF as their rehabilitation service; 273 (26.4%), SNF; and 736 (71.0%), HHA.

**Table 1.** Characteristics of Study Population of Older Adults

Characteristic	Participants, No. (%) (N = 1036)
Age, y	
65-69	63 (6.1)
70-74	130 (12.6)
75-79	172 (16.6)
80-84	237 (22.9)
85-89	218 (21.0)
≥90	216 (20.9)
Women	670 (64.7)
Race/ethnicity	
White	685 (66.1)
Black	252 (24.3)
Hispanic	54 (5.2)
Other	22 (2.1)
NA or missing	23 (2.2)
Marital status	
Not married	729 (70.4)
Married	305 (29.4)
NA or missing	2 (0.2)
Highest level of education	
<High school diploma	329 (31.8)
High school diploma	289 (27.9)
>High school diploma	393 (37.9)
NA or missing	25 (2.4)
Rehabilitation setting	
HHA	736 (71.0)
IRF	27 (2.6)
SNF	273 (26.4)
Health status	
Excellent	58 (5.6)
Very good	155 (15.0)
Good	310 (29.9)
Fair	334 (32.2)
Poor	177 (17.1)
NA or missing	2 (0.2)
Health conditions^a^	
Heart attack	276 (26.6)
Heart disease	367 (35.4)
Hypertension	802 (77.4)
Arthritis	761 (73.5)
Osteoporosis	302 (29.2)
Diabetes	359 (34.7)
Lung disease	250 (24.1)
Stroke	266 (25.7)
Dementia	186 (18.0)
Cancer	306 (29.5)

Abbreviations: HHA, home health agency; IRF, inpatient rehabilitation facility; NA, not applicable; SNF, skilled nursing facility.

^a^ Data for health conditions were missing in 0 to 7 participants per condition.

### Correlation of Summary Assessments

**Figure 1.**
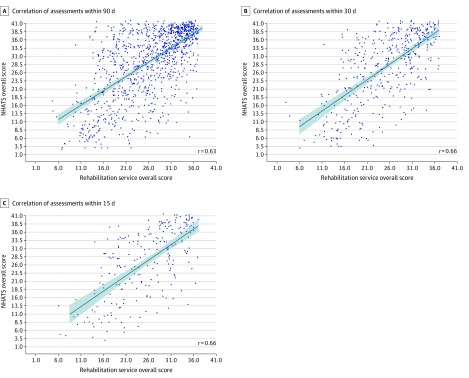
Scatterplots of Correlation of the National Health and Aging Trends Study (NHATS) Overall Score With Rehabilitation Service Overall Score

The mean (SD) rehabilitation service functional score was 27.5 (7.2) compared with 30.5 (10.1) for the NHATS assessment. The median (interquartile range) time between assessments was 37 (18-61) days. The correlation coefficient of the 2 assessments was 0.63 (95% CI, 0.59-0.66) (Figure 1). The correlation increased to 0.66 (95% CI, 0.60-0.71) among the 429 individuals with NHATS assessments within 30 days of the rehabilitation assessment and to 0.66 (95% CI, 0.58-0.72) among the 230 individuals with NHATS assessments within 15 days of the rehabilitation assessment. Scatterplots of the functional assessment scores are displayed in Figure 1. Visual inspection of the plots indicated a balanced distribution across the range of scores, with the possible exception of individuals with lower rehabilitation functional scores. At the low end of rehabilitation scores, relatively few individuals had NHATS scores above the correlation line, although only a small number of individuals had low rehabilitation scores overall. By facility setting, the correlations were 0.57 (95% CI, 0.49-0.65) for SNF, 0.64 (95% CI, 0.59-0.67) for HHA, and 0.74 (95% CI, 0.50-0.87) for IRF (eTable 5 in the Supplement).

In the linear regression model adjusting for days between assessments, the rehabilitation assessments had a strong association with the NHATS assessments (β = 0.87; 95% CI 0.80 to 0.93). Days since rehabilitation setting assessment was associated with a small increase in the NHATS assessment score (β = 0.02; 95% CI, 0.00 to 0.04). The model intercept was 5.73 (95% CI, 3.77 to 7.70), and the overall *R*^2^ was 0.40 (Table 2). In the model that added rehabilitation setting, the association of the rehabilitation assessment with the NHATS assessments increased (β = 1.00; 95% CI, 0.93 to 1.08). The SNF setting assessment was associated with an increase in the disability score compared with the HHA setting (β = 4.02; 95% CI, 2.72 to 5.31), the intercept decreased to 0.72 (95% CI, −1.79 to 3.24), and the *R*^2^ increased slightly to 0.42.

**Table 2.** Association of Rehabilitation Setting Functional Assessment Score and Other Factors With National Health and Aging Trends Study Function Assessment^a^

Factor	β (95% CI)	
	Model A	Model B
Rehabilitation function score	0.87 (0.80 to 0.93)	1.00 (0.93 to 1.08)
Rehabilitation setting		
HHA	NA	1 [Reference]
SNF	NA	4.02 (2.72 to 5.31)
IRF	NA	−2.54 (−5.51 to 0.42)
Days since rehabilitation discharge	0.02 (0.00 to 0.04)	0.03 (0.01 to 0.05)
Intercept	5.73 (3.77 to 7.70)	0.72 (−1.79 to 3.24)
*R*^2^	0.40	0.42

Abbreviations: HHA, home health agency; IRF, inpatient rehabilitation facility; NA, not applicable; SNF, skilled nursing facility.

^a^ Model A was used to evaluate the correlation of the assessments adjusting for days between the assessments. Model B was used to evaluate for differences explained by rehabilitation setting. In addition, Model B provided a formula that can be used to standardize functional scores across rehabilitation settings.

### Agreement of Summary Assessments

**Figure 2.**
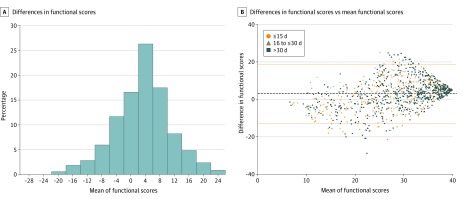
Plots of Differences in NHATS and Rehabilitation Service Functional Scores Differences in functional scores were calculated as NHATS scores − rehabilitation service scores.

The mean (SD) difference of the rehabilitation service scores (NHATS score − rehabilitation score) was 2.96 (7.91). Plots of the differences in the scores are displayed in Figure 2. The histogram of differences is consistent with a normal distribution (Figure 2A). The Bland-Altman plot of the differences in scores compared with the mean of the score showed that only 59 of 1036 individuals (5.7%) had a difference in function scores that was more than 2 SDs of the mean difference (Figure 2B). Scores on NHATS were slightly higher than rehabilitation service scores at the high end of mean function scores. A relatively small number of individuals had low mean function scores (156 of 1036 individuals [15.1%] with mean scores ≤20). Among individuals with mean scores of 20 or less, there was a low frequency of individuals with NHATS scores greater than rehabilitation scores, particularly from the HHA setting, which had 64 individuals in this range (Figure 2; eFigure 2 in the Supplement). We did not observe a pattern of variation in differences by days between assessments (Figure 2B).

### Correlation of Individual Components

Mean scores and correlations of the 6 individual disability components by assessment time difference are displayed in Table 3. The correlations of the individual components were all statistically significant and typically in the 0.45 to 0.55 range (eg, toileting: *R*^2^ = 0.47; 95% CI, 0.42-0.52; bathing: *R*^2^ = 0.46; 95% CI, 0.41-0.51; dressing: *R*^2^ = 0.52; 95% CI, 0.43-0.62). The correlations either remained stable or slightly increased as the days between assessments narrowed (eg, bathing at ≤30 days: *R*^2^ = 0.42; 95% CI, 0.34-0.50; at ≤15 days: *R*^2^ = 0.44; 95% CI, 0.33-0.54). The eating assessment consistently had the lowest correlation, ranging from 0.34 to 0.37 (≤90 days: *R*^2^ = 0.37; 95% CI, 0.31-0.42; ≤30 days: *R*^2^ = 0.36; 95% CI, 0.27-0.44; ≤15 days: *R*^2^ = 0.34; 95% CI, 0.22-0.45).

**Table 3.** Summary Data of Functional Scores From NHATS and Medicare Rehabilitation Assessments, Including Correlation of These Assessments Based on Days Since Rehabilitation Discharge Assessment

Assessment	Score at 90 d (n = 1036)^a^			Score at 30 d (n = 429)			Score at 15 d (n = 230)		
	Mean (SD)		*R*^2^ (95% CI)	Mean (SD)		*R*^2^ (95% CI)	Mean (SD)		*R*^2^ (95% CI)
	NHATS	Rehab		NHATS	Rehab		NHATS	Rehab	
Summary functional capacity	30.5 (10.1)	27.5 (7.2)	0.63 (0.59-0.66)	28.9 (10.5)	26.4 (7.3)	0.66 (0.60-0.71)	28.3 (10.3)	26.8 (7.2)	0.66 (0.58-0.72)
Individual item functional capacity									
Eating	6.2 (1.6)	5.4 (0.9)	0.37 (0.31-0.42)	6.1 (1.7)	5.4 (1.0)	0.36 (0.27-0.44)	6.1 (1.7)	5.4 (0.9)	0.34 (0.22-0.45)
Toileting	5.4 (1.8)	4.7 (1.5)	0.47 (0.42-0.52)	5.2 (2.0)	4.5 (1.6)	0.5 (0.42-0.57)	5.0 (2.0)	4.6 (1.6)	0.51 (0.41-0.60)
Bathing	4.4 (2.4)	3.6 (1.5)	0.46 (0.41-0.51)	4.2 (2.4)	3.4 (1.4)	0.42 (0.34-0.50)	4.0 (2.4)	3.5 (1.4)	0.44 (0.33-0.54)
Dressing	5.0 (2.3)	4.4 (1.6)	0.52 (0.43-0.62)	4.6 (2.3)	4.2 (1.6)	0.55 (0.48-0.61)	4.5 (2.3)	4.3 (1.6)	0.57 (0.48-0.65)
Transfers	5.7 (2)	4.7 (1.2)	0.45 (0.40-0.50)	5.3 (2.2)	4.5 (1.3)	0.49 (0.41-0.56)	5.2 (2.1)	4.5 (1.3)	0.44 (0.33-0.54)
Walking	3.9 (2.6)	4.7 (1.8)	0.44 (0.39-0.49)	3.6 (2.5)	4.5 (1.9)	0.50 (0.43-0.57)	4.5 (1.8)	3.4 (2.5)	0.50 (0.39-0.59)

Abbreviations: NHATS, National Health and Aging Trends Study; rehab, rehabilitation.

^a^ Data were missing in 0 to 8 individuals (0%-0.08%) per item.

## Discussion

Our study of more than 1000 individuals found that functional assessments in rehabilitation settings are correlated with criterion-standard research assessments. The assessments also had good overall agreement. These findings provide important new evidence to support the use of routine care assessments as a functional outcome measure in some contexts. Given that these measures can be linked to Medicare claims, the range of potential applications of these measures is broad.

The correlation of the 2 assessments performed within 90 days was in the range of what is considered moderate by some and substantial by others.^15,16,17^ The magnitude of correlation suggests that the 2 assessments measure a similar underlying construct of function, which ranges from an independent to a dependent status. We also found that the correlation of the assessments increased slightly as the time between assessments narrowed from 90 days to 30 days. This finding supports construct validity, given that we expected function to change somewhat after rehabilitation, with some people continuing to improve while others experience some worsening.

The analysis of the differences of the scores found a small bias for higher NHATS scores overall and a normal distribution of differences with few outliers. The slightly higher scores in NHATS could be in part because of a difference in the scaling of items in NHATS compared with SNF and HHA, considering that the NHATS scale for each functional item was up to 7 points for individuals who are independent and do not use devices, whereas the functional scales were mostly truncated at 6 points for SNF and HHA.^12^ Therefore, a fully independent person who does not use devices would score a 42 in NHATS but a 37 in SNF or HHA. Another potential reason for differences in scores is that the NHATS functional scores were obtained by self-report whereas function in rehabilitation services were rated by staff observing the patient. We were not able to determine with our data which approach was a more accurate overall measure of function.

While the overall NHATS scores were slightly higher than rehabilitation scores, we found that most individuals with lower mean function scores had NHATS assessments lower than rehabilitation assessments, particularly when compared with HHA assessments. The reason for this finding is not certain. First, only 156 individuals (64 from HHA) had lower mean function, so it is possible that the differences at the lower mean range were random selection error. Other possibilities are that individuals with lower mean scores may have had conditions that rendered them more susceptible to functional worsening after rehabilitation (eg, degenerative disorders) or conditions associated with lower self-perception of dependence (eg, depression) compared with independent staff ratings. Future studies are needed to evaluate how reasons for rehabilitation and comorbidities influence agreement in the scales.

Functional assessments, a highly patient-centered measure, are challenging to incorporate into longitudinal studies. Therefore, the Medicare data makes it feasible to better understand function and the factors associated with it among older adults. The assessments could be used in observational studies linked to Medicare or potentially as a clinical trial outcome. Based on our findings, it would be appropriate to use data from rehabilitation settings in large studies like ours with either a similar distribution of function or populations with moderate to high function. We recommend caution using this data in populations with low function until we have a better understanding of why rehabilitation scores are typically higher than NHATS scores in individuals with lower mean function scores.

### Strengths and Limitations

The primary strengths of our study were rehabilitation assessments from routine care, a large sample, and a rigorous criterion standard. Including data from all 3 rehabilitation settings was another strength. Correlations for each setting were in the moderate range. By including all settings, we were able to derive an equation that can be used to standardize functional scores across sites.

Our study has important limitations in addition those already noted. We were only able to select FIM components that overlap in topics and scoring scales with the NHATS assessment. We had relatively few assessments from IRF after applying our exclusions. Because NHATS dates are limited to month and year, our difference in days could be off by up to 30 days. Our analysis was limited to discharge assessments from rehabilitation services. Therefore, we were not able to make conclusions about the validity of admission and interim assessments.

It is important to note that, as of fiscal year 2020, the FIM assessments will no longer be performed in IRFs. The assessment was replaced by the quality indicators in the Quality Reporting Program (QRP). Based on the considerable overlap of FIM and the QRP items, the FIM was dropped to reduce administrative burden. Although a separate validation of the NHATS with QRP-graded function would be preferred, it seems likely that that QRP measures will also generally correlate with criterion-standard measures of function given the similarity of FIM and QRP items. The major difference in FIM and QRP items is in the scaling of the items. Compared with the 7-point scales on the FIM, the QRP has 6-point scales because it does not consider the use of a device. The QRP categories also have a slight difference in what is considered supervision and the percentage of effort a helper provides to perform the activity. The QRP items are specifically used as quality metrics and therefore bias in scoring is also a concern, as it was with the FIM.

## Conclusions

This study extends our knowledge of the validity of rehabilitation functional assessments in the routine care setting. Our results demonstrated that these data were correlated and agree with a rigorous criterion-standard functional assessment. These findings suggest that rehabilitation facility assessments provide sufficiently accurate estimates of function for application in many research contexts.

## Notes

Supplement. **eAppendix. **Supplementary Methods **eTable 1. **Disability Assessment Instruments by Rehabilitation Setting **eTable 2. **Crosswalk Scheme for Eating, Bathing, and Transfers Bed **eTable 3. **Crosswalk Scheme for Toileting Hygiene and Dressing **eTable 4. **Crosswalk Scheme for Walking and Mobility **eTable 5. **Summary Data of Individual Component and Overall Functional Scores From NHATS and Medicare Rehabilitation Assessments and the Correlation of These Assessments **eFigure 1. **Flow Chart **eFigure 2. **Bland-Altman Plot of Differences in Functional Scores (NHATS Minus Rehabilitation Service Scores) vs the Mean of the Functional Scores by Rehabilitation Service **eReferences.**
