# Supplementary material for: Validation of Medicare Rehabilitation Functional Assessments in Routine Care
Source: JAMA Netw Open. 2020 May 13;3(5):e204702. doi: 10.1001/jamanetworkopen.2020.4702 (PMC7221510; doi:10.1001/jamanetworkopen.2020.4702)
Supplement: Supplement. — eAppendix. Supplementary Methods eTable 1. Disability Assessment Instruments by Rehabilitation Setting eTable 2. Crosswalk Scheme for Eating, Bathing, and Transfers Bed eTable 3. Crosswalk Scheme for Toileting Hygiene and Dressing eTable 4. Crosswalk Scheme for Walking and Mobility eTable 5. Summary Data of Individual Component and Overall Functional Scores From NHATS and Medicare Rehabilitation Assessments and the Correlation of These Assessments eFigure 1. Flow Chart eFigure 2. Bland-Altman Plot of Differences in Functional Scores (NHATS Minus Rehabilitation Service Scores) vs the Mean of the Functional Scores by Rehabilitation Service eReferences. [file jamanetwopen-3-e204702-s001.pdf]

Supplementary Online Content

Kerber KA, Skolarus LE, Feng C, Burke JF. Validation of Medicare rehabilitation functional assessments in routine care. *JAMA Netw Open*. 2020;3(5):e204702.
doi:10.1001/jamanetworkopen.2020.4702

eAppendix. Supplementary Methods
eTable 1. Disability Assessment Instruments by Rehabilitation Setting
eTable 2. Crosswalk Scheme for Eating, Bathing, and Transfers Bed
eTable 3. Crosswalk Scheme for Toileting Hygiene and Dressing
eTable 4. Crosswalk Scheme for Walking and Mobility
eTable 5. Summary Data of Individual Component and Overall Functional Scores From NHATS and Medicare Rehabilitation Assessments and the Correlation of These Assessments
eFigure 1. Flow Chart
eFigure 2. Bland-Altman Plot of Differences in Functional Scores (NHATS Minus Rehabilitation Service Scores) vs the Mean of the Functional Scores by Rehabilitation Service
eReferences.

This supplementary material has been provided by the authors to give readers additional information about their work.

© 2020 Kerber KA et al. *JAMA Network Open.*

eAppendix. Supplementary Methods

Supplementary Methods:
In the IRF setting, the FIM items are scored 1-7 with higher scores representing greater independence (eTables 1, 2, 3).[1] To score each item, rehabilitation staff are instructed to consider the individual's actual performance of the activity and then select the lowest functional score within any 24-hour period from the 3-day period encompassing the day of discharge to two calendar days prior. Based on our six FIM components, the overall functional score ranges from 7 to 49.

MDS items are scored on a 5-point scale and have been previously crosswalked to FIM items for all of our variables except mobility/walking (eTable 1).[2] Staff score MDS items based on functioning over the previous up to 7 day period.[3] For the crosswalk of mobility/walking assessment in MDS to the relevant FIM item, we used the MDS's locomotion on unit self-performance variable (eTable 3).

OASIS functional assessment instrument questions are similar to the MDS questions however there are slight differences in the item response scales so that the applicable crosswalk translates to a 4-6 point scale depending on the item (eTables 1, 2, 3).[4,5]

© 2020 Kerber KA et al. *JAMA Network Open.*

eTable 1. Disability Assessment Instruments by Rehabilitation Setting

Assessment Tool	Rehabilitation Setting
Functional Independence Measure (FIM™)	Inpatient Rehabilitation Facility (IRF)
Minimum Data Set (MDS)	Skilled Nursing Facility (SNF)
Outcome and Assessment Information Set (OASIS)	Home Health Agency (HHA)

The FIM mark is owned by UB Foundation Activities, Inc. (UBFA, Inc.).

© 2020 Kerber KA et al. *JAMA Network Open.*

eTable 2. Crosswalk Scheme for Eating, Bathing, and Transfers Bed
SNF column indicates MDS scores crosswalked to FIM scores; HHA column indicates OASIS scores crosswalked to MDS crosswalked to FIM.

NHATS	IRF	SNF	HHA
7. No help, no device	7. Complete independence		
6. No help, yes device, no difficulty	6. Modified independence	0=6	0=0=6
5. No help, yes device, yes difficulty (a little, some, or a lot)	5. Supervision or set up		
4. Yes help, independent most of the time	4. Minimal assistance	1=4.5	1=1=4.5
3. Yes help, independent sometimes	3. Moderate assistance	2=3.5	2[a,b]=2=3.5
2. Yes help, independent rarely	2. Maximal assistance	3=2.5	3=3=2.5
1. Yes help, independent never or missed activity	1. Total assistance<br>0. Activity does not occur	4/7/8=1	4/5/[6][c]=4/7/8=1

NHATS = National Health and Aging Trends Study; IRF = inpatient rehabilitation facility; SNF= skilled nursing facility; HHA = home health agency; FIM = functional independence measure; MDS = minimum data set; OASIS = Outcome and Assessment Information Set.
SNF items: Eating, G0110H1; Bathing, G0120A; Transfer bed, G0110B1
HHA items: Eating, M1870; Bathing, M1830; Transfer bed, M1850.
[a] Eating item from HHA, level 2 = unable to feed self and must be assisted or supervised throughout the meal/snack.
[b] Transfer item from HHA, level 2= able to pivot and bear weight but unable to transfer self.
[c] Only bathing had 6 categories.

© 2020 Kerber KA et al. *JAMA Network Open.*

eTable 3. Crosswalk Scheme for Toileting Hygiene and Dressing
SNF column indicates MDS scores crosswalked to FIM scores; HHA column indicates OASIS scores crosswalked to MDS crosswalked to FIM.

NHATS	IRF	SNF	HHA
7. No help, no device	7. Complete independence		
6. No help, yes device, no difficulty	6. Modified independence	0=6	0=0=6
5. No help, yes device, yes difficulty (a little, some, a lot)	5. Supervision or set up		
4. Yes help, independent most of the time	4. Minimal assistance	1=4.5	1=1=4.5
3. Yes help, independent sometimes	3. Moderate assistance	2=3.5	
2. Yes help, independent rarely	2. Maximal assistance	3=2.5	2=3=2.5
1. Yes help, independent never or missed activity	1. Total assistance<br>0. Activity does not occur	4/7/8=1	3=4=1

NHATS = National Health and Aging Trends Study; IRF = inpatient rehabilitation facility; SNF= skilled nursing facility; HHA = home health agency; FIM = functional independence measure; MDS = minimum data set; OASIS = Outcome and Assessment Information Set.
SNF items: Toileting hygiene, G0110I1; Dressing, G0110G1.
HHA items: Toileting hygiene, M1845; Dressing, M1810, M1820.

© 2020 Kerber KA et al. *JAMA Network Open.*

eTable 4. Crosswalk Scheme for Walking and Mobility
SNF column indicates MDS scores crosswalked to FIM scores; HHA column indicates OASIS scores crosswalked to MDS crosswalked to FIM.

NHATS	IRF	SNF	HHA
7. No help, no device	7. Complete independence		0=7
6. No help, yes device, no difficulty	6. Modified independence	0 (no cane or walker) =6	0=6
5. No help, yes device, yes difficulty (a little, some, a lot)	5. Supervision or set up	0 (yes, cane or walker)= 5	2=0=5
4. Yes help, independent most of the time	4. Minimal assistance	1=4	
3. Yes help, independent sometimes	3. Moderate assistance	2=3	
2. Yes help, independent rarely	2. Maximal assistance	3=2	3=3=2
1. Yes help, independent never or missed activity	1. Total assistance<br>0. Activity does not occur. Bed rest or Wheel chair	4/7/8=1	4/5/6=4/7/8=1

NHATS = National Health and Aging Trends Study; IRF = inpatient rehabilitation facility; SNF= skilled nursing facility; HHA = home health agency; FIM = functional independence measure; MDS = minimum data set; OASIS = Outcome and Assessment Information Set.
NHATS item derived from mobility outside questions. IRF item: Locomotion: walk.
SNF items: Locomotion on unit, G0110E; Use of cane, G0600A; Use of walker; G0600B, use of cane. Toileting hygiene, G0110I1; Dressing, G0110G1.
HHA items: Toileting hygiene, M1845; Dressing, M1810, M1820.

© 2020 Kerber KA et al. *JAMA Network Open.*

eTable 5. Summary Data of Individual Component and Overall Functional Scores From NHATS and Medicare Rehabilitation Assessments and the Correlation of These Assessments

	90-day (N=1,036)[a]		
	NHATS	Rehabilitation	Correlation (95% CI)
Inpatient Rehabilitation Facility, N=27			
Eating	6.4 (1.6)	6.1 (0.8)	0.15 (-0.25, 0.50)
Toileting	5.0 (2.2)	5.0 (1.4)	0.45 (0.08, 0.71)
Bathing	4.5 (2.5)	4.6 (1.1)	0.63 (0.32, 0.81)
Dressing	4.7 (2.6)	4.3 (1.5)	0.57 (0.24, 0.78)
Transfers	5.3 (2.4)	5.0 (1.1)	0.64 (0.33, 0.81)
Walking	3.2 (2.5)	4.5 (1.8)	0.59 (0.26, 0.79)
Summary	29.1 (11.1)	29.4 (6.6)	0.74 (0.50-0.87)
Skilled Nursing Facility, N=273			
Eating	5.8 (1.9)	4.9 (1.1)	0.40 (0.30, 0.50)
Toileting	4.8 (2.1)	3.3 (1.2)	0.37 (0.27, 0.47)
Bathing	3.6 (2.4)	2.6 (1.1)	0.33 (0.22, 0.43)
Dressing	4.3 (2.4)	3.3 (1.1)	0.46 (0.36, 0.55)
Transfers	5.0 (2.3)	3.5 (1.2)	0.44 (0.34, 0.53)
Walking	3.1 (2.5)	3.1 (1.4)	0.39 (0.28, 0.48)
Summary	26.5 (10.9)	20.7 (5.6)	0.57 (0.49-0.65)
Home Health Agency, N=736			
Eating	6.4 (1.4)	5.6 (0.7)	0.30 (0.23, 0.36)
Toileting	5.7 (1.6)	5.3 (1.3)	0.49 (0.43, 0.54)
Bathing	4.7 (2.3)	3.9 (1.5)	0.45 (0.39, 0.50)
Dressing	5.2 (2.2)	4.8 (1.5)	0.51 (0.46, 0.56)
Transfers	5.9 (1.8)	5.1 (1.0)	0.40 (0.33, 0.45)
Walking	4.2 (2.5)	5.3 (1.6)	0.41(0.35, 0.47)
Summary	32.0 (9.3)	30.0 (6.1)	0.63 (0.59-0.67)

[a] Data was missing in 0-8 individuals (0%-0.08%) per item.

© 2020 Kerber KA et al. *JAMA Network Open.*

eFigure 1. Flow Chart

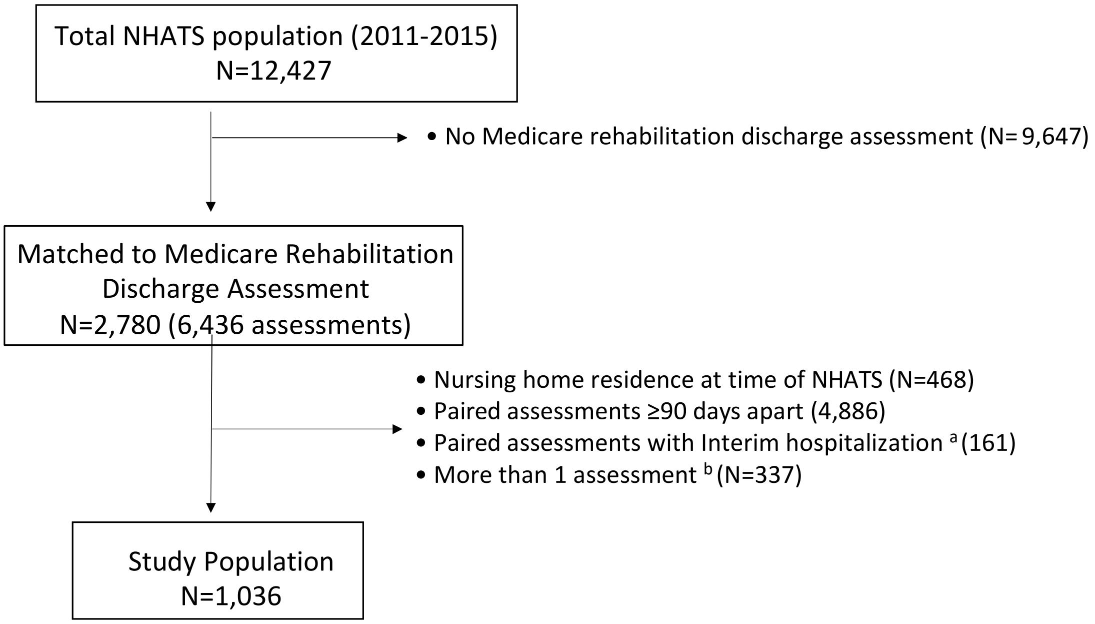

[a] Hospitalization that occurred in between the rehabilitation assessment and the NHATS assessment.
[b] When an individual had >1 eligible assessment, the assessment with the lowest days difference was used.

© 2020 Kerber KA et al. *JAMA Network Open.*

eFigure 2. Bland-Altman Plot of Differences in Functional Scores (NHATS Minus Rehabilitation Service Scores) vs the Mean of the Functional Scores by Rehabilitation Service

A) all rehabilitation services; B) Inpatient rehabilitation facility; C) Skilled nursing facility; D) Home health agency.

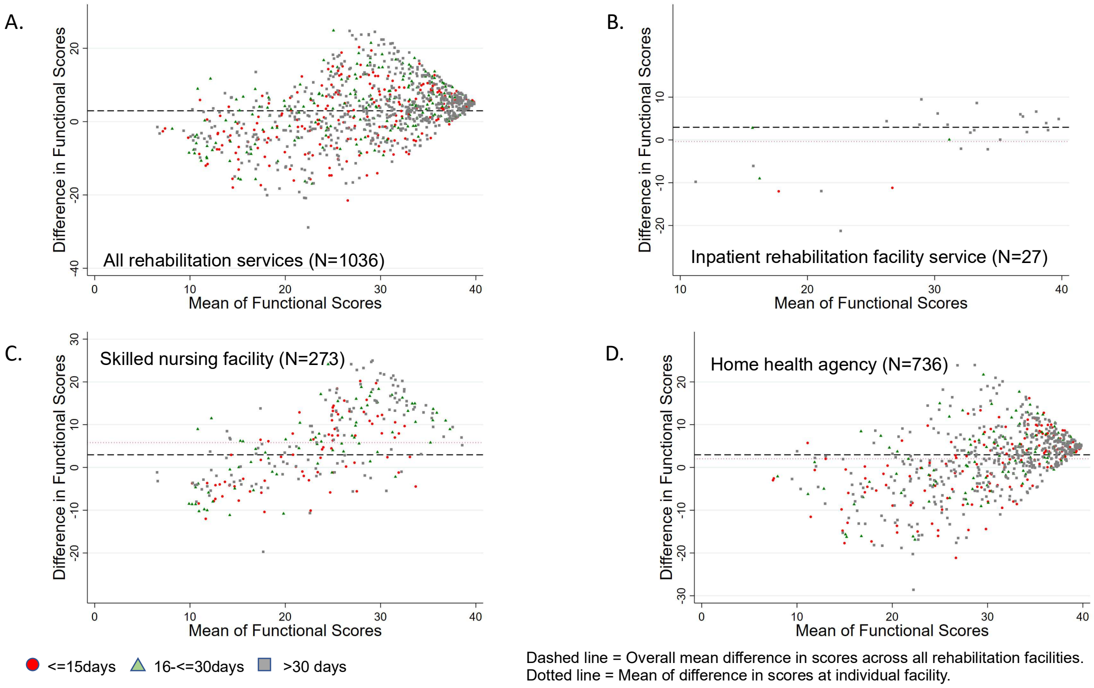

Dashed line = Overall mean difference in scores across all rehabilitation facilities.
Dotted line = Mean of difference in scores at individual facility.

© 2020 Kerber KA et al. *JAMA Network Open.*

eReferences.

1. The inpatient rehabiliation facility - patient assessment instrument (IRF-PAI) training manual: Effective 10/01/2012. Centers for Medicare & Medicaid Services. Accessed at: https://www.cms.gov/Medicare/Medicare-Fee-for-Service-Payment/InpatientRehabFacPPS/Downloads/IRFPAI-manual-2012.pdf.
2. Williams BC, Li Y, Fries BE, Warren RL. Predicting patient scores between the functional independence measure and the minimum data set: development and performance of a FIM-MDS "crosswalk". *Archives of physical medicine and rehabilitation.* 1997;78(1):48-54
3. Minimum data set (MDS) - Version 3.0. Accessed at: https://www.cms.gov/Medicare/Quality-Initiatives-Patient-Assessment-Instruments/NursingHomeQualityInits/Downloads/Archive-Draft-of-the-MDS-30-Nursing-Home-Comprehensive-NC-Version-1140.pdf.
4. Morris JN, Jones RN, Fries BE, Hirdes JP. Convergent validity of minimum data set-based performance quality indicators in postacute care settings. *Am J Med Qual.* 2004;19(6):242-247
5. Outcome and assessment information set (OASIS-D) guidance manual. Centers for Medicare and Medicaid Services. Accessed at: https://www.cms.gov/Medicare/Quality-Initiatives-Patient-Assessment-Instruments/HomeHealthQualityInits/Downloads/OASIS-D-Guidance-Manual-final.pdf.

© 2020 Kerber KA et al. *JAMA Network Open.*
